# Clinical Research Practitioner: Transformation to a Competent Professional Through Reflective Practice

**DOI:** 10.1111/nhs.70009

**Published:** 2024-12-04

**Authors:** Ibiyemi Sadare

**Affiliations:** ^1^ University of West London London UK

**Keywords:** clinical research practitioners, competency, organization, professionalism, reflection, reflective practice

## Abstract

The National Institute for Health and Care Research (NIHR) has significantly contributed to the growth of clinical research activities in the United Kingdom. Central to this expansion is the pivotal role of Clinical Research Practitioners (CRPs) within the healthcare organization. This narrative explores reflective practice as a dynamic tool and essential requirements for their transformation and development into competent CRPs. Additionally, this article adds to the growing literature to highlight the benefits of having a professionally registered CRP for NHS organizations. By weaving together personal insights, professional development, and the integration of acquired knowledge, the narrative illuminates the profound impact of reflective practice on the evolution of a skilled and adept CRP. This review adds to the current literature on the importance of reflective practice in improving professionalism and competency for registered CRPs. The narrative emphasizes lifelong learning and reflective practice integration in clinical research training programs. It suggests further research on workplace reflection for knowledge integration. This resource is valuable for CRPs, organizations, and policymakers, emphasizing professionalism, competency development, and reflective practice in clinical research.

**Summary**

- This article provides insight into the evolving role of CRPs and emphasizes reflective practice as a crucial tool for their transformation and development into competent professionals.
- It also highlights the benefits for organizations of having professionally registered CRPs, providing recommendations for organizations to invest in training CRPs to improve their competency, confidence, and professionalism.
- For organizations to focus on role advancement and specialization opportunities to allow CRPs to take on more challenging tasks and contribute significantly to research activities.

## 1 Introduction

National Institute for Health and Care Research (NIHR), since its inception in 2006 as the health research arm of NHS, has aimed to make a meaningful difference in people's lives through the research they fund (NIHR 2024). Since then, there has been a gradual increase in clinical research activities across the United Kingdom and the research workforce within the NHS (Faulkner‐Gurstein, Jones, and McKevitt 2019). Clinical Research Practitioners (CRPs) are one of the most widely used research workforces for conducting studies with the participants, and they occupy a crucial position in supporting the delivery of clinical research in healthcare (NIHR 2023).

The CRP role presents opportunities for organizations wanting to be part of the research landscape to contribute to recruitment data toward the NIHR vision (Faulkner‐Gurstein, Jones, and McKevitt 2019). CRPs have a range of educational backgrounds as the foundation of their practice in the valuable work they do to support research delivery in different settings (Sonstein and Jones 2018). They need to become professionally recognized and be on the Professional Standards Authority (PSA) accredited register. However, as CRP registration is relatively new, many organizations have yet to have established structures to support this fast‐growing network of research‐oriented professionals. Therefore, the CRP must be proactive in self‐development.

So far, very little is known about some functional and transformational skills a CRP needs to support professional development. This narrative explores reflective practice as a dynamic tool and essential requirements for their transformation and development into competent CRP professionals.

### 1.1 Importance of Competent CRPs in Raising the Organizational Research Profile

Burns (2019), p. 49 defines professionalism as “the attitude, approach and practices that go with a profession as a group; or the characteristic/s of a professional person, if considering an individual.” He explains that professionalism refers to a profession's membership, and the individual professional must act according to the profession's ethos. The concept of professionalism refers to membership in a profession. Professionalism does not have a fixed meaning, but it means different things to different people (Fox 1992; Evans 2008; Evetts 2013).

In healthcare, a multidisciplinary team (MDT) consists of specialists from various professions working together to coordinate patient care. Individual professionalism is crucial for team's effectiveness, increasing with experience (Griffith 2017; Wynd 2003). The Health Professions Council (2012) emphasizes the importance of organizations in supporting professional development and upholding values. Understanding workforce needs is crucial for a professionally driven culture, and organizations should provide skills and development opportunities. CRPs are an important part of the MDT in secondary care settings. As clinical trials are becoming more complex, NIHR understands the role and importance of CRPs as essential in ensuring that clinical trials are successful. Therefore, the role of CRPs is evolving, but with challenges (Faulkner‐Gurstein, Jones, and McKevitt 2019). Additionally, the role is increasingly demanding that CRPs are skilled in digital solutions, real‐time data entry, e‐prescribing support, and biological sample processing. Managing role complexity without a proper professional development plan becomes more challenging.

Gee and Cooke (2018) emphasize the role of practitioners in research capacity development, withCRPs playing a crucial role in this transformation. CRPs must be hands‐on in achieving recruitment targets, contributing to patient care and raising organizational profile. To ensure competency, employers and sponsors require formal professionalization of the CRP role in healthcare research. NIHR and the Academy for Healthcare Science (AHSC) have empowered CRPs to become part of the PSA‐accredited register in the United Kingdom.

### 1.2 Professionalism in Clinical Trial Practice

The modern NHS is constantly evolving, necessitating changes in healthcare practices. Evetts (2013) notes that professionalism is dynamic and subject to change; therefore, professional CRPs should possess the skills to adapt to these changes. Benefits of maintaining a professional practice for organizations include high‐quality clinical research delivery standards, high proficiency and reliability of the CRP workforce, public protection, and the integration of skilled professionals into MDT.

CRP professionals perform a variety of roles in clinical trial delivery; typical roles in research practices involve, information giving, communication, and coordinating. Centrally, their attitudes and behaviors are under a microscope for scrutiny by the trial participants, their family members, the trial management key contacts, and the organization. Therefore, professionalism in clinical trial practice is underpinned by these attributes, which are knowledge base, specialized skills, duty of care, a range of competencies based on a defined framework, standards of proficiency, and a code of conduct, which comprises elements related to integrity, confidentiality, and other behavioral aspects. The role of a professional body in regulating the CRP profession cannot be over‐emphasized; compliance with these established standards ensures the individual becomes proficient in the discharge of the expected duties. The conduct should reflect the codes as given, attitudes and actions in practice to enable the CRPs to act professionally, as guided by the expected standards of practice.

**FIGURE 1:**
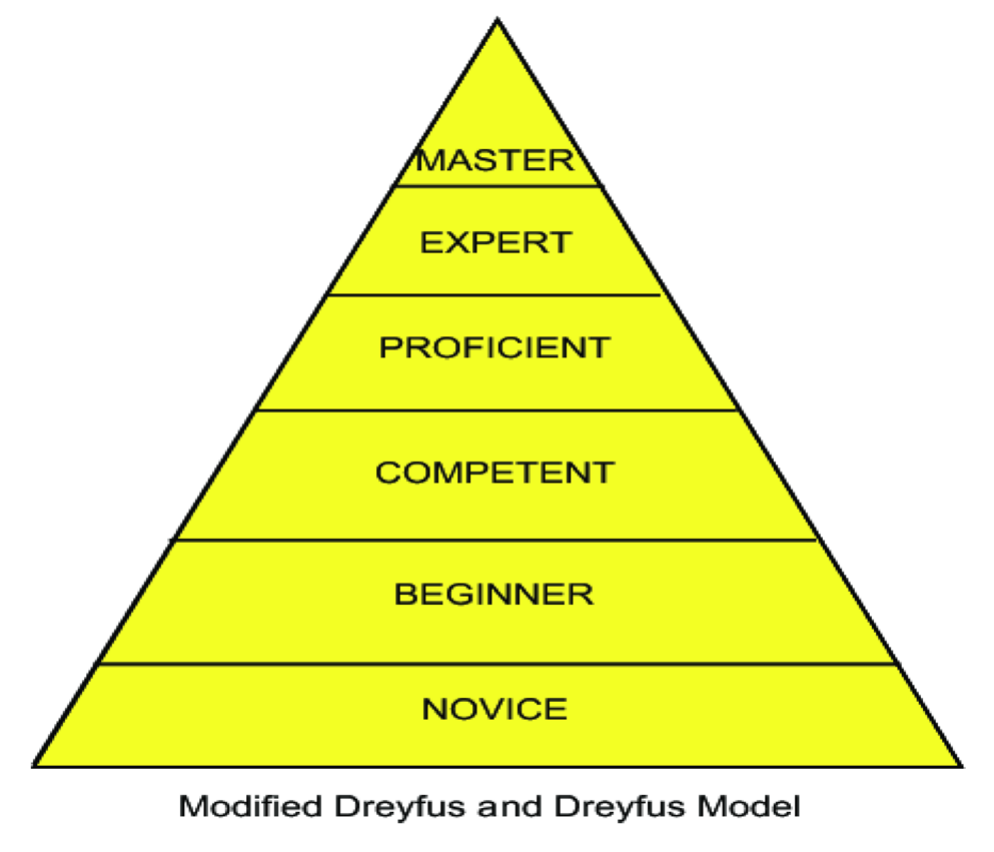
Modified Dreyfus and Dreyfus skill acquisition model (Satava Richard and Gallagher Anthony 2015).

Skills, knowledge, and behavior characterize a good model of professionalism. Dreyfus and Dreyfus (1980) model of skill acquisition (Figure 1) (Satava Richard and Gallagher Anthony 2015) shows how an individual progresses through five levels based on experience: novice, advanced beginner, competent, proficient, and expert. These progressions are seen through the assessment of knowledge, skills, and behavior.

Peña (2010) notes that the acquisition of skills is a factual learning process, while the Dreyfus model identifies as a factual–conceptual model, this tends to demonstrate how diverse skills are acquired. According to this model, the competency framework for CRPs demonstrates how professionalism can be improved in practice by adherence to the regulatory, ethical (International Council for Harmonization of Technical Requirements for Pharmaceuticals for Human Use (ICH) Guidelines and the Declaration of Helsinki), and professional code of practice.

### 1.3 Competency Development

The competency, values, behavior, and practice challenges can impact a CRP's professional practice. Professionalism evolves as the CRP moves from different proficiency levels in clinical research practice. Novice (otherwise referred to as the self‐starters), intermediate, and competent practitioner phases are three distinct phases comparable to the first three phases in the Dreyfus model (Figure 1). These phases are challenging before the transformation from the novice to the competent phase, but skill acquisition helps to overcome the challenging circumstances. Most CRPs would admit that self‐development impedes the transition to becoming an autonomous and confident practitioner. Equally, mastery for the competent CRP is gained by self‐motivation, which stems from identifying the learning needs, seeking opportunities to learn, and putting the learning into practice.

Admittedly, one cannot exclude the importance of organizational support through the line manager, who supports the self‐ and professional development of the CRP. A vital support is provided in terms of development through identifying the learning needs of new starters and those who have been in the post for some time. Therefore, line managers help to set objectives for completion of competencies relevant to individual CRP roles and scope of practice. By employing a competency framework and supporting tools such as the NIHR integrated workforce framework (IWF), CRPs can identify gaps, evaluate their current skills, and track their progress (AHSC 2022).

The NIHR Research Delivery Network (formerly Clinical Research Network) developed the IWF using the joint task force clinical research core competency framework (JTF framework) (Sonstein et al. 2014) and Royal College of Nursing Framework for Clinical Research Nurses (2011). The IWF framework, which includes three key components: clinical research, clinical context, and leadership aims to describe the CRP role and identify opportunities for development. The IWF is a tool used by CRPs to structure career development and acknowledge progression. It helps identify roles and their expansion beyond their remit and helps map roles within the team. The IWF also helps identify skills gaps and creates a strategic approach to workforce planning. Managers can use the framework to facilitate discussions with staff about personal development and career progression (NIHRIWF 1 2017).

### 1.4 The Academy for Healthcare Science (AHSC) Maintains a PSA Accredited Register

AHCS in the United Kingdom is the central body overseeing and representing the entire UK Healthcare Science (HCS) workforce. It works collaboratively with healthcare professional bodies, life science industry professionals, and CRPs. The AHCS aims to increase visibility and recognition of these diverse workforces by promoting their contributions to healthcare and ensuring their roles are acknowledged within the broader healthcare system. (AHSC 1, no date). Additionally, it seeks to represent the profession in authoritative responses to relevant organizations and work toward establishing a statutory register for all healthcare scientists. The UK HSC workforce is regulated through an accredited PSA register, providing support to the registrants, ensuring patient safety, and raising the profession's profile. AHSC set up registration procedures for CRPs since 2020 (AHSC 1 n.d.); any applicant deemed to meet the required standards of proficiency will hold accredited registration. The core behavior, skills, and knowledge required to be a clinical research professional are defined by the AHSC. The CRPs are autonomous and accountable professionals who deliver clinical and health‐related research, having the authority to make decisions and act accordingly with their professional knowledge base.

Registration requires applicants to demonstrate safe practice under the expected scope of practice and code of conduct. This ensures trial participants' rights, safety, and well‐being, and ensures high‐quality, compassionate care. Furthermore, it assures safe and compassionate care to a high standard and the confidence to challenge others not practising to that expected level. The scope of practice defines personnel, knowledge, and conduct, while the standard of practice defines the clinical context. Competencies are linked and assessed through portfolios of evidence, appraisal, feedback, reflection, and re‐validation.

Developing expertize in research practice is essential for any CRP looking to expand their scope of practice in clinical and health‐related research delivery. Apart from formal education, evidence of training and skill competency is key to gaining professional registration status. To be included on the register, CRPs must possess significant experience and knowledge, most likely after the first year of working at the practitioner level. The standard of proficiency is provided as a guide for the expected code of conduct, behaviors, skills, and knowledge. Competency assessment before joining the register is achieved in‐house through the line manager, who manages the competency workbook, feedback from other professionals that the CRP works with and a portfolio of evidence.

The applicant must submit three reflective writings for professional accountability, working across boundaries, and leadership application. These reflective activities serve as a retrospective account of the CRP experience and lessons learned about the CRP scope of practice and standards of proficiency, particularly to given standards peculiar to the experience (AHSC 2 n.d.). The minimum standards expected for registration with the AHCS are set out in the 16 standards of proficiency, which are grouped according to (1) professional responsibility (standards 1–6) and (2) behavior, knowledge and skills based on clinical research (standards 7–10), clinical context (standards 11–14), and leadership (standards 15–16).

Interestingly, the 16 standards ties in with the JTF framework to account for the elements of The International Council for Harmonisation of Technical Requirements for Pharmaceuticals for Human Use (ICH), good clinical practice (GCP), and Declaration of Helsinki. Structurally, IWF (Table 1) is based on three domains namely clinical research, clinical context, and leadership. Depending on the role and the expected responsibility and skill, each domain has four levels: awareness, core, intermediate, and advanced. The clinical research domain in the IWF is defined by seven elements that align with the International Joint Task Force Harmonized Core Competency Framework for the Clinical Research Professional. The clinical context domain consists of four elements that focus on the patient care pathway and people's involvement in clinical research, which may include direct care delivery. The leadership domain consists of a single element that directs users to the NHS leadership model and framework, which supports consistent leadership development across health and care positions (NIHRIWF 2 2017).

**TABLE 1:** An extract from the NIHR Clinical Research Network Integrated Workforce Framework Working Table V2.1 (NIHR 2018).

Domains	Element	Element definition
Clinical research domain	Ethics	Underpin the conduct of clinical research activity, patient safety, and clinical governance
	Research development and regulation	Encompasses the relevant regulatory frameworks governing clinical research
	Science and research design	The pursuit and application of knowledge and understanding following a systematic methodology based on evidence
	Clinical studies operations	Encompasses the processes, responsibilities, and systems required to facilitate efficient, safe, and participant‐focused research
	Study and site management	Involves the infrastructure and resources required to assess, arrange, and deliver a research study
	Data management and informatics	Quality process for capture of data as part of delivery for a study and analysis, which enables the research outcomes of the study to be determined
	Communication and research relationships	Actively seek to work across boundaries and involve investment to maintain and enhance research delivery, including NHS engagement across all clinical specialities and care settings
Clinical context domain	Clinical governance	Accountability for continuous improvement in the quality of services and safeguarding high standards of care in an environment where clinical care flourishes
	Patient care pathway	A predictable clinical course, involving multidisciplinary management, in which the different tasks by the professionals involved are defined, optimized and sequenced
	Clinical communication	Person‐centered communication that respects and values concerns, ideas, expectations, needs, and feelings in any health condition and according to social or cultural context
	Clinical care	Direct delivery of care to the patient by the health or care professional
Leadership domain	Leadership	The leadership element points to the developing people—improving care (DPIC) framework, the NHS Leadership Academy, NHS England, and resources of the National Improvement and Leadership Development Board, which guide a consistent approach to leadership development for staff in health and care and highlight the principles that are relevant for application across the research workforce

The AHSC's requirements for CRP registration emphasize the importance of academic knowledge as the foundation for self‐development. Academic knowledge imparts skills like analytical, critical thinking, reflection, creativity, originality, active learning techniques, and complex problem‐solving (Wellington and Sikes 2006). This knowledge is crucial for practitioners to engage in thoughtful action and advance in practice (Lester 2004; Thompson and Pascal 2012).

CRP professionals rely on academic knowledge and develop skills in clinical trial coordination through training opportunities relating to the specific disease area, research delivery principles, and leadership. They aim to become autonomous practitioners, skilled in recruiting participants, and adhere to recognized standards of practice and proficiency.

Reflection and reflexivity are crucial in bridging the gap between academic knowledge and professional experience. The developmental process is circular, blending skills from applied research to practice quality improvements (Nonaka, Toyama, and Byosiere 2001) as cited by (Nilsen, Nordström, and Ellström 2012). Reflection is central to translating learning to practice and managing both learning types in a structured, systematic way to confidently practice professionally.

## 2 Reflective Process

Reflection is a learning tool whereby one can learn from experience (Jasper 2013; Pitsoe and Maila 2013). It is a process of thoughts captured and critically analyzed to improve, inform, and change the practice. Combined with the identified learning style, one can build on the practice reflectively using just one or a combination of models for improvement. Schön (1983) broadly describes the development of professional knowledge through reflection and explains it with these two concepts:

Reflection‐in‐action refers to the reflective thinking one does while doing the action, shown by experience.

Reflection‐on‐action is reflective thinking after the experience, thinking about what to do differently next time, and processing thoughts creatively for increased competency.

Critical thinking and understanding can be gained through experiences, transforming into learning, enabling better future choices, and responses through in‐depth thinking and new insights concerning self or practice (Scanlan, Care, and Udod 2002; Nilsen, Nordström, and Ellström 2012). Jarvis (1992) draws attention to a deeper meaning of reflective thinking as “that form of practice that seeks to problematize many situations of professional performance so that they can become potential learning situations, and so the practitioners can continue to learn, grow and develop in and through practice.” In other words, it is a way of systematically merging different unstructured practice methods to achieve a more formalized approach to work. The professional experience is the foundation for learning and development; the practitioner purposefully incorporates reflection, so, in essence, bridging the gap between underlying theory and practice.

Nilsen, Nordström, and Ellström (2012) maintain that reflection is a workplace mechanism that can be used to integrate practice‐based knowledge and research‐based knowledge for greater productivity. It is a thoughtful process for consciously analyzing the practice using theories in practice. Schon's reflection‐on‐action concept has been widely used retrospectively as a predictive activity for practice development.

### 2.1 Key Theories of Reflective Practice

Different established models exist in the literature to support the reflective practitioner in accurately reflecting practice, viewing it from different angles. It is a continuous process, which does not stop at a definitive point, hence the cyclical nature of most of them. Some common ones are Driscoll's what model (Driscoll 2007, p. 43; Bulman and Schutz 2013, p. 234), the experience, learning, and reflection (ERA) cycle (Jasper 2013), whereby arrows link experience, learning, and reflection together triangularly; Kolb's experiential learning cycle (Kolb 1984) based on theories about the learning process through experience (concrete experience, reflective observation, abstract conceptualization, and active experimentation). Gibbs (1988) added more stages with specific directions, an example of a single loop because of the lack of critical challenge assumptions of self and organization. There are six stages, with cues at each stage to help draw out more experience: experience, feeling, evaluation, analysis, conclusion, and action plan.

**FIGURE 2:**
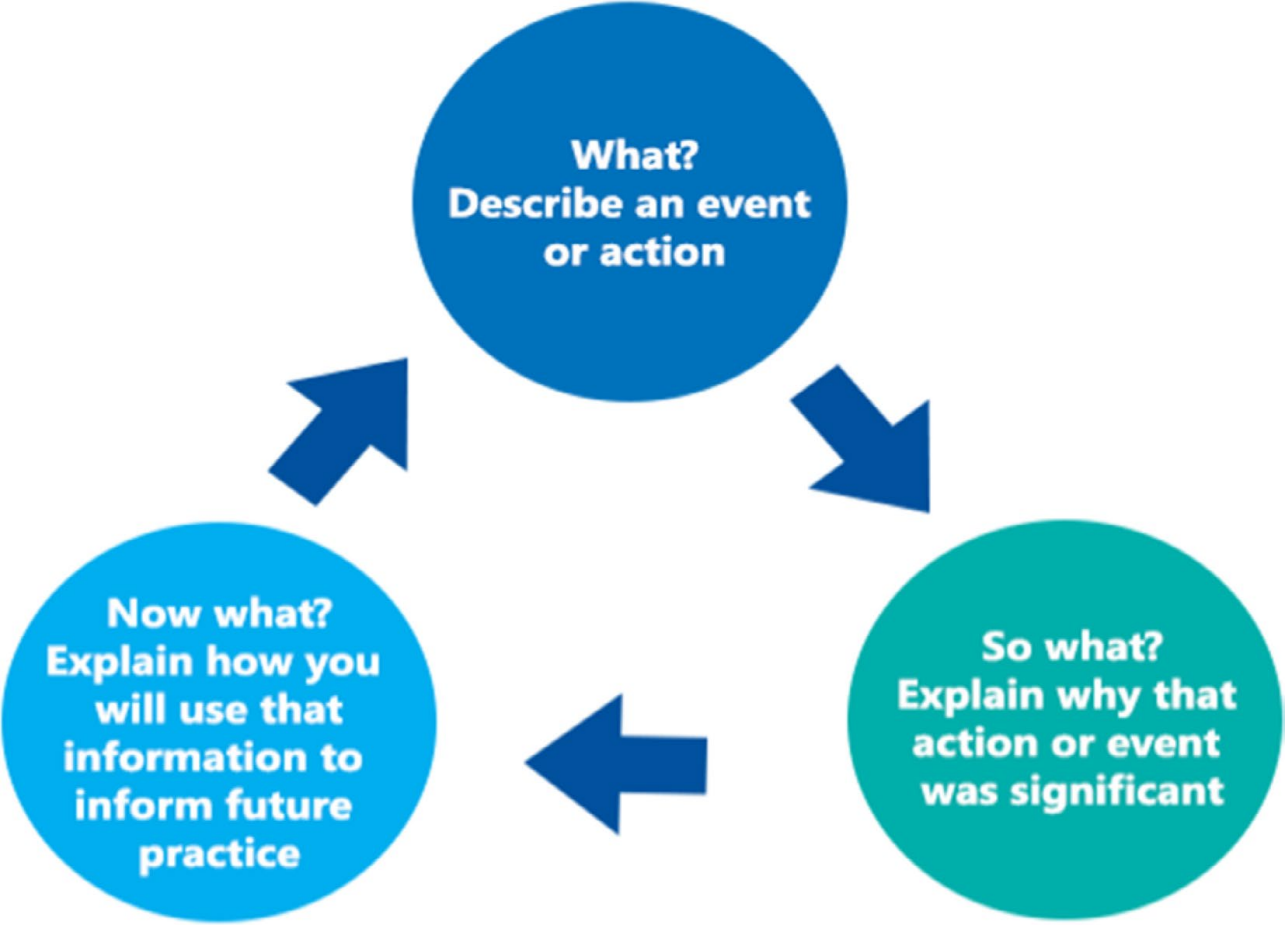
Driscoll's model of reflection (Nursing Answers 2018).

Driscoll's what model (Figure 2): Driscoll developed a simple model in the mid‐1990s as an expansion of Terry Borton's model in the 1970s (Borton, 1970). The Driscoll model is based on these three essential questions, what? so what?, and now what? The model analyses the user's experience, fully describing the situation and reflecting on the experience by asking “what” happened? The “So what” stage helps to analyze the issue, examine the implications of the situation and are the available information to guide the decision to a more desirable outcome. The last step is “Now what?” which technically helps to think of alternatives and action plans and the consequences of the action (Jasper 2013, p. 100).

Of course, Driscoll's models have key benefits: it is reasonably easy to use, especially for a novice practitioner, and it is easy to work through the questions and adapt to real‐life cases (Jasper 2013, pp. 99–100). The author also maintains that the model enables reflections toward a change in practice or toward self‐development with the three main questions (Fook 2013, p239). Critics question the ability of Driscoll's model to provide a deeper, meaningful reflection. However, it is possible if the user engages with the three stem questions beyond a superficial level (Forrester 2020).

### 2.2 Reflection Model to Improve Practice—A Case Study Detailing a CRP Personal Reflection

My career in clinical trials practice has spanned over 20 years, including supporting the delivery of NIHR‐funded research. My research experience has been beneficial in expanding an extensive portfolio of studies and increasing clinical trial participant recruitments within the Trust. A key challenge to the practice was the difficulty in navigating the clinical pathway to recruit patients to oncology clinical trials in a structural way due to the clinical pathway of patient management. The issue was not with the cancer diagnostic pathway, but the treatment pathway, which varies depending on the type of planned treatment due to available infrastructure. Often, patients are transferred to neighboring hospital Trusts for radiotherapy and surgical and systemic therapy treatments. My team do miss the patients for recruitment during the inter‐hospital transfer.

Driscoll's model of reflection guided my reflective process to strategically work out a solution to the issues that were important and pivotal to my practice. This model is easy for me because of the ease of transformation from one level to another. The model is centered on three basic questions in the case study below:

**Table:** 

**Case study: A challenging situation that developed my understanding of (a) an organizational boundary**
**What** A colorectal trial faced a significant challenge in recruiting participants, with only two patients enrolled in the first year and no new recruits by the second year. This was concerning as my team had specific recruitment targets we failed to meet. A retrospective review revealed that patients had already been enrolled in the treatment hospital, not ours. We identified that the core issue was the patient treatment pathway, which hindered our ability to recruit patients for the trial. Reflecting on this experience, it was a moment of realization about the structural and procedural barriers that can impede research. It underscored the importance of understanding how institutional pathways impact recruitment efforts and the need for proactive problem‐solving to avoid failure.
**So what** I realized that to address the situation, a new strategy was essential. After analyzing the patient pathway, I identified the critical failure point in how we approached patients for recruitment. I decided to involve the recruiting team from the treatment hospital to explore alternative solutions, but I initially hesitated, unsure if they would accept my proposal for accrual sharing or if it would be feasible. Despite my uncertainty, I anticipated a response and knew that taking the initiative was necessary to move forward.
**Now what** This reflection highlights a leadership‐driven approach to implementing effective change in a clinical research setting. I initiated a collaborative process by involving key stakeholders from both hospitals, presenting the challenge with clear data, and facilitating a practical solution. By presenting the potential patient recruitment figures, I effectively communicated the need for change. Although direct modifications to our local pathway were challenging, we agreed on the most feasible and the best option—collaborating with the other hospital to manage patient referrals and data sharing, leading to increased patient accrual. This experience taught me the value of working across professional and organizational boundaries. It fostered a deeper understanding of how expansion can be driven by collaboration and mutual support. The process allowed me to develop critical leadership skills such as negotiation, cross‐team integration, and adaptability. This challenge served as an opportunity for personal and professional growth, transforming my perspective on leadership. With the desire to see effective changes, the experience reinforced the importance of fostering organizational change through open communication, and teamwork while leveraging diverse cultural perspectives. Overall, I embraced this challenge to cultivate skills necessary for driving cultural transformation, highlighting that true collaboration can link boundaries, align goals, and ultimately achieve positive outcomes.

“Now what” enabled me to reflect on how this experience has improved my practice. Understanding the importance of working across boundaries has helped me to open complex trials, open more communication channels among the stakeholders, improve performance through root cause analysis and solve issues that are the barriers to functioning effectively. I have used boundary‐spanning techniques to bring multiple groups together in a new direction to cross boundaries and discover new possibilities. There are innovations through diversity, trust is built, and there is improvement in the team's performance. I got feedback from different groups to improve and empower colleagues and myself, with increased autonomy to practice and confidence to collaborate with others to foster good working relationships.

Professionally, there is an improvement in my work by being open to change, encouraging honest conversation, and providing equal opportunities for collaboration. I shared my concerns with different teams, utilizing their specialist knowledge to achieve a good outcome. I understand the process of escalating situations to higher authorities for effective solutions; alternatively, we would have deemed the trial a failure and closed it to avoid wasting resources pursuing it.

### 2.3 How Did I Know It Worked and How Did I Use This Learning From the Reflection to Inform Future Practice?

The success indicators included increased patient enrolment in clinical trials and heightened staff engagement. Interestingly, staff from other Trusts were eager to collaborate with us to improve the research experience for patients. The clinicians became more engaged in opening communication channels to improve research activities. The Driscoll framework facilitated the examination of practice changes to ensure support for new trials during the planning stage. Reflective processes helped identify and overcome barriers to research delivery within the Trust, encouraging a review of current practices against similar approaches. Key to these changes were developing resilience, staying focused despite resistance, and maintaining an open, honest, and confident learning attitude. The clinicians became more engaged in opening communication channels to improve research activities. With the Driscoll framework, I can now explore issues in ongoing practices to ensure adequate support for upcoming trials before they begin.

The reflective process has helped to identify and address barriers to delivering research within the Trust to improve current practices. The successful implementation of highlighted changes requires resilience, focus, honesty, confidence, and open‐mindedness. Time constraints and communication breakdowns were challenges, but forward planning, and early engagement with stakeholders within the organization helped mitigate them. Reflection is crucial for practice, allowing for assessment and learning from experiences. It consolidates knowledge, competence, and confidence, and builds confidence (Nilsen, Nordström, and Ellström 2012). Initially, the thought process was dominated by organizational failure, but reflection helped navigate it toward solutions for service and practice improvement. It is also imperative to collaborate with colleagues to improve work practices and use creative thinking skills.

## 3 Conclusion

### 3.1 Future Directions and Recommendations

‐Emphasizing the importance of lifelong learning.

As CRPs are the link that connects the participants with the research, they must be able to navigate limitations and weaknesses that could be barriers in practice. Expectations from the employing organization demand a need to be open to new experiences, understand a person‐centered approach and be more creative in building evidence to support their practice. One way of achieving this is to critically reflect, explore and understand leading research in this area and its application to health care. In terms of career development, there are now opportunities from NIHR for a registered CRP to access funding to pursue their research interests, create new ideas and generate improvements in research skills that can be transferable to my work practices as well as part of the solution to global challenges in clinical trials recruitment. In addition, to have a more profound knowledge toward becoming an independent and autonomous practitioner, one can develop more skills (both intellectual and transferable) for career development as this will be used to integrate information from current developments to have an impact on the practice (Sonstein and Jones 2018).

‐Suggestions for integrating reflective practice into training programs.

Reflective writing is new to CRPs until they are about to register, this presents a challenge. The narrative describes the usefulness of a reliable method of writing a reflective piece to support registration application. Clinical research is expanding rapidly to other unfamiliar territories as the health need rises associated with the economic burden of providing solutions for healthcare commissioners. Therefore, CRP personnel need to work toward professionalism and be equipped to take on the major role. It is also key that organizations invest in training this valuable workforce to improve their competency, confidence and empowerment, fulfilling their role among the other healthcare professionals in the organization.

CRPs are already accessing different training courses, but many still need reflective practice. Practice educators within the organizations can develop frameworks for learning and competency frameworks incorporating reflective practice. One crucial way of supporting the development of a self‐starter CRP toward their development is incorporating reflections in annual appraisals and one‐to‐one discussions. The CRPs came from different educational backgrounds with no formal training in reflective practice, which is one of the necessary skills for registration. However, if organizations provide support early enough through the line managers, these professionals will be empowered to develop into autonomous practitioners (Nonaka 1994).

The rationale for this narrative is to consolidate evidence on the importance of professionalism and to increase understanding of the benefit to CRPs and the employing organizations. With trials becoming complex, CRPs are increasingly delegated more responsibilities, extending beyond the traditional screening and randomizing participants to clinical trials. Considerably more work will need to be done to determine evidence of how workplace reflection can be used to integrate knowledge into practice. The organizations and the workforce development team need to draw on resources to incite CRPs toward registration, maintain their registration, and keep them in the profession. Further work is needed to design a defined developmental career structure for CRPs. This imperative arises from the recognition that the role of CRPs is dynamic and ever‐changing, necessitating a career framework that accommodates and promotes their development. Clarity in career structure is critical for providing CRPs with a clear idea of the expectations, responsibilities, and opportunities available at various stages of their career. A well‐defined structure would describe the development from a novice to an advanced practitioner, providing CRPs with direction and purpose as they navigate their professional journey.

Recognizing the different abilities, interests, and aspirations of CRPs would help their professional ambitions with a flexible career framework. Furthermore, a focus on role advancement toward advanced practice demonstrates a dedication to identifying and utilizing the complete range of CRPs' abilities. By providing more education, training, and specialization possibilities, CRPs can take on increasingly challenging tasks and substantially contribute to patient care. This would allow them to develop their career in clinical research, research management, and academia.

Collaboration between healthcare institutions, educational bodies, and professional associations is paramount to achieving these goals. Establishing mentorship programs, continuing education initiatives, and creating clear criteria for advanced practice roles can contribute to the overall success of the career structure for CRPs. Regular reviews and updates to the structure can ensure its relevance in an ever‐changing healthcare landscape.

### 3.2 Relevance for Clinical Practice

This comprehensive exploration serves as a valuable resource for CRPs, organizations, and policymakers, highlighting the significance of professionalism, competency development, and reflective practice in the ever‐evolving landscape of clinical research. CRPs must be committed to lifelong learning that can foster the competencies to help in transforming into competent and confident professionals.

## Author Contributions

**Ibiyemi Sadare:** conceptualization, writing – original draft, writing – review and editing, investigation.

## Ethics Statement

The author has nothing to report.

## Conflicts of Interest

The author declares no conflicts of interest.

## Data Availability

Data sharing not applicable to this article as no datasets were generated or analysed during the current study.
